# Association of Genetic Polymorphisms in STAT 3, STAT 5b and GWAS Identified *PTPN22* Gene with Rheumatic Heart Disease

**DOI:** 10.1186/1755-8166-7-S1-P110

**Published:** 2014-01-21

**Authors:** Usha Gupta, Avshesh Mishra, Saurabh S Rathore, Snober S Mir, SK Agarwal, Naveen Garg, Balraj Mittal

**Affiliations:** 1 Department of Genetics, Sanjay Gandhi Postgraduate Institute of Medical Sciences (SGPGIMS), Lucknow, UP, India; 2 Department of Biotechnology, Integral University, Lucknow, UP, India; 3 Department of Cardiovascular and Thoracic Surgery, Sanjay Gandhi Postgraduate Institute of Medical Sciences (SGPGIMS), Lucknow, UP, India; 4 Department of Cardiology, Sanjay Gandhi Postgraduate Institute of Medical Sciences (SGPGIMS), Lucknow, UP, India

## Background

Rheumatic heart disease (RHD) is an inflammatory, autoimmune disease, occurring as a consequence of group A streptococcal infection complicated by rheumatic fever (RF). Cytokines are important mediators of inflammatory and immune responses. JAK-STATs have been demonstrated to be critical elements in signaling by certain families of cytokines. GWAS has identified *PTPN22* SNPs as non-HLA genetic variants to be associated with susceptibility to autoimmune diseases. Based on these, we looked for association of genetic variants of *STAT 3*, *STAT 5B* and GWAs identified *PTPN22* with RHD in North Indian population.

## Methods and results

This case-control study included 400 RHD patients and 200 controls. The polymorphisms were identified using RFLP/Taqman probes. Statistical analysis was performed by using SPSS. We observed that *STAT3* CG and GG genotypes were significantly associated with RHD (p=0.024 & p=0.027 respectively), *STAT5b* CT&TT genotypes were significantly associated with RHD (p=0.001 & p=0.002 respectively) while both the SNPs of *PTPN22* gene did not show any association with RHD. Further categorization of RHD patients into mitral valve disease (MVD) and combined valve disease (CVD) subgroups revealed that *STAT3* CG&GG genotypes were associated with MVD and *STAT5b* CT&TT genotypes were also associated with both MVD&CVD.

## Conclusions

*STAT3* &*STAT5b* gene polymorphisms may play an important role in the pathogenesis of RHD but GWAS identified *PTPN22* SNPs may not be associated with susceptibility of RHD.

